# Expression, tandem repeat copy number variation and stability of four macrosatellite arrays in the human genome

**DOI:** 10.1186/1471-2164-11-632

**Published:** 2010-11-15

**Authors:** Deanna C Tremblay, Graham Alexander, Shawn Moseley, Brian P Chadwick

**Affiliations:** 1 Department of Biological Sciences, Florida State University, King Life Science Building, Tallahassee, FL 32306-4295, USA; 2 IGSP Sequencing Core Facility, Duke University, Durham, NC 27708, USA

## Abstract

**Background:**

Macrosatellites are some of the largest variable number tandem repeats in the human genome, but what role these unusual sequences perform is unknown. Their importance to human health is clearly demonstrated by the 4q35 macrosatellite D4Z4 that is associated with the onset of the muscle degenerative disease facioscapulohumeral muscular dystrophy. Nevertheless, many other macrosatellite arrays in the human genome remain poorly characterized.

**Results:**

Here we describe the organization, tandem repeat copy number variation, transmission stability and expression of four macrosatellite arrays in the human genome: the TAF11-Like array located on chromosomes 5p15.1, the SST1 arrays on 4q28.3 and 19q13.12, the PRR20 array located on chromosome 13q21.1, and the ZAV array at 9q32. All are polymorphic macrosatellite arrays that at least for TAF11-Like and SST1 show evidence of meiotic instability. With the exception of the SST1 array that is ubiquitously expressed, all are expressed at high levels in the testis and to a lesser extent in the brain.

**Conclusions:**

Our results extend the number of characterized macrosatellite arrays in the human genome and provide the foundation for formulation of hypotheses to begin assessing their functional role in the human genome.

## Background

More than half of the human genome is composed of repetitive DNA [1], a proportion of which are tandem repeats. Tandem repeats are characterized by a given DNA sequence being repeated immediately adjacent to and in the same orientation as the first. In almost all cases the number of repeat units at a given locus vary between individuals and as such they are more commonly known as variable number tandem repeats (VNTRs).

Macrosatellites are among the largest VNTRs in the human genome [2] and are characterized by individual repeat units of several kilobases (kb) and tend to be specific to a location on one or two chromosomes. Among the human macrosatellites, the best characterized are RS447, an array of 20-103 4.7 kb tandem repeat units on 4p16.1 along with a few copies at 8p23 [3], D4Z4 on 4q35 and 10q26, an array of as few as 1 to over 100 3.3 kb tandem repeat units [4,5] and DXZ4, an array of 50-100 copies of a 3-kb repeat unit specifically located at Xq23-24 [6]. The role of some tandem repeat DNA is more obvious due to their location, such as the extensive alpha satellite tandem repeats that define human centromeres [7] or the tandem repeat DNA at human telomeres [8]. What function macrosatellites have in genome biology is unclear. However, reduction in the size of D4Z4 on 4q35 to fewer than 10 tandem repeat units is associated with onset of the third most common inherited form of muscular dystrophy, facioscapulohumeral muscular dystrophy (FSHD) [5]. Numerous models have been proposed to explain how D4Z4 contraction could result in FSHD [9]. One early model suggested that reduction in the size of D4Z4 packaged as heterochromatin could result in a position-effect on adjacent gene expression [10]. Results from several studies testing this model are mixed, with some showing support [11-14], and others not [15-19]. More recently focus has turned to expression of the array itself. Like RS447, that contains an open reading frame (ORF) in each monomer that codes for a novel deubiquitinating enzyme [20], an ORF is present in D4Z4 monomers that encodes double homeobox 4 (DUX4) [10], a DNA binding protein [21] that alters expression levels of myogenic regulators in myoblasts [22]. DUX4 expression can be detected in patient myoblasts [21] but not control individuals. Recent evidence suggests that detectable levels of DUX4 in patients is due to polyadenylation and stabilization of transcripts originating from the most distal monomer in the array due to a polyadenylation signal located immediately distal to the array [23], a feature that is found only on chromosomes with the FSHD pathogenic 4qA161 haplotype [24].

The X-linked macrosatellite DXZ4 is also expressed [25] and contains several short ORFs [6], but none show any homology to known proteins. Unlike RS447 and D4Z4, the location of DXZ4 on the X chromosome means that in females it is exposed to the mammalian dosage compensation process X chromosome inactivation (XCI). XCI repackages the chosen inactive X chromosome (Xi) into facultative heterochromatin [26]. Somewhat counter intuitively, DXZ4 adopts a more euchromatic conformation on the Xi while the surrounding chromosome is packaged into heterochromatin, whereas on the active X chromosome (Xa) DXZ4 is packaged into heterochromatin surrounded by euchromatin [25,27]. The euchromatic Xi form of DXZ4 is bound by the epigenetic organizer protein CTCF [28] suggesting that on the Xi, DXZ4 is performing a different function than on the Xa. Intriguingly, contracted D4Z4 arrays in FSHD adopt a chromatin configuration similar to DXZ4 on the Xi [29] including the binding of CTCF [30] that likely contributes to the disease permissive state.

These data suggest that tandem repeat copy number, chromatin organization and expression are important features of macrosatellites, and provide some insight into functional attributes of these sequence, in particular the impact of short arrays in FSHD. Indeed, in *Saccharomyces cerevisiae*, retention of large rDNA tandem repeats are necessary for sister chromatid cohesion and maintenance of genome integrity [31]. Therefore exploring macrosatellite VNTR and organization in the human genome is a high priority. In the current manuscript we describe the organization, expression, tandem repeat copy number polymorphism and stability of four macrosatellite arrays in the human genome.

## Results

### Characterization of the TAF11-Like macrosatellite array on chromosome 5p15

In order to identify novel human macrosatellite arrays, the UCSC genome browser http://genome.ucsc.edu/ was used with the segmental duplication annotation activated to explore by eye the complete human genome sequence (NCBI36/hg18). The segmental duplication track identifies duplications of 1 kb or greater that share 90% or more DNA sequence identity [32]. Regions annotated as segmental duplications that originate from the same chromosomal interval and displayed a regular repeat masking pattern were selected for further examination. These rough criteria were chosen based on the fact that the well characterized macrosatellite arrays DXZ4, D4Z4 and RS447 were clearly identified using the same parameters.

**Figure 1:**
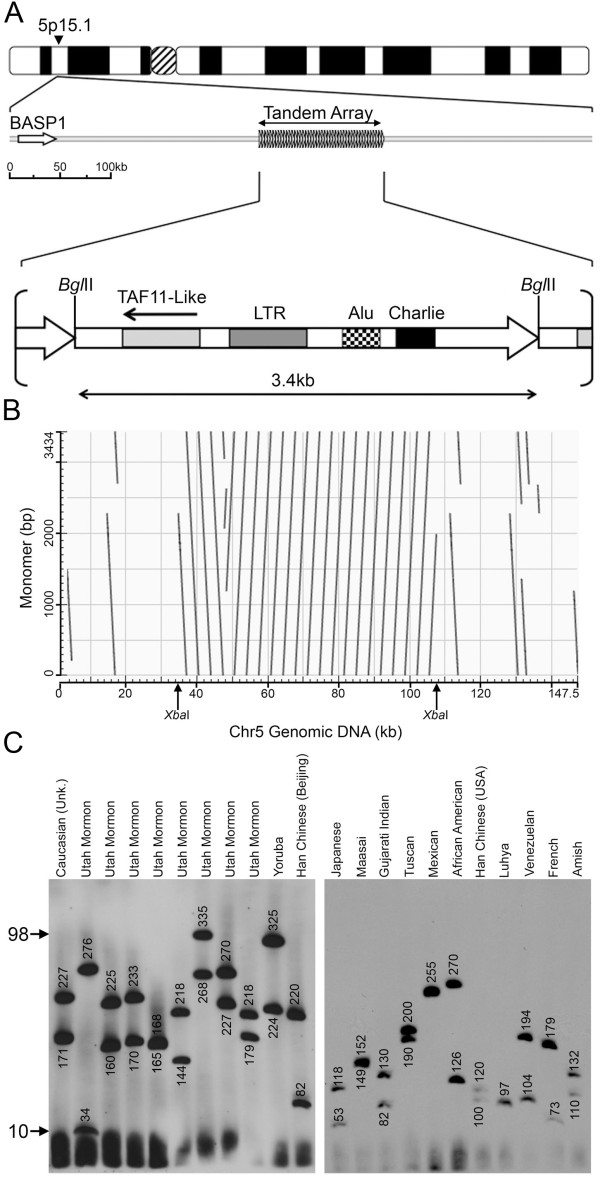
**Characterization of the Chromosome 5p15.1 TAF11-Like macrosatellite array**. (A) Ideogram of chromosome 5 and schematic map of the region surrounding the macrosatellite. Right facing arrow heads represent the array. *BASP1 *is indicated to the left. Scale indicates the approximate distances represented on the map in kb. Below the Array is a map of an individual 3.4 kb monomer (Large right facing arrow). BglII restriction endonuclease sites that define a single monomer are indicated to the left and right. Repetitive elements LTR, Alu and Charlie are indicted by the shaded and patched boxes. The ORF for *TAF11-Like *is highlighted and orientation indicated by the left facing arrow. (B) Higher-order organization of the chromosome 5 array as revealed by dot-plot analysis. On the y-axis is a 3.4 kb monomer and on the x-axis is the 147 kb genomic interval (17.5 Mb according to build hg18). XbaI restriction endonuclease sites that immediately flank the array that are used for the PFGE are indicated. Dot-plot generated using NCBI Blast, and output image labelled in Adobe Photoshop CS2. (C) Copy number variation of the chromosome 5 macrosatellite array. Southern blot of XbaI cut DNA from 22 unrelated individuals separated by pulsed field gel electrophoresis and hybridized with a chromosome 5 macrosatellite probe. The ethnicity of the individuals is indicated at the top. The size of the hybridizing fragments in kb are indicated on the blot. Arrows to the left indicate the range in size of the array as inferred copy number.

One tandem array that spans ~143 kb (Chr5: 17,543,001 - 17,686,000 of hg18) was identified on the short arm of human chromosome 5 at 5p15.1. The closest annotated gene to the 5p15.1 macrosatellite repeat is *BASP1 *(Brain Abundant, membrane attached Signal Protein) located approximately 210 kb distal to the array (Figure 1A). On the proximal side, the nearest annotated gene *CDH18 *(CaDHerin 18 type 2 preproprotein) is located over 1.8 Mb away. A cluster of expressed sequence tags (ESTs) represented by BC028204 (UniGene cluster Hs.407197) resides approximately 160 kb proximal to the array with some EST members as close as 30 kb.

An individual repeat unit of the array is approximately 3.4 kb and is defined by a BglII restriction enzyme recognition site that cuts once per monomer (Figure 1A). Monomers within the contiguous array share between 99.3-99.7% nucleotide identity based on BLAT alignment results http://genome.ucsc.edu using monomers from genome build hg18, with most variation due to single nucleotide polymorphisms (SNPs) between monomers. The base composition of each monomer is approximately 49.6% GC and a little under a third (31.7%) of each monomer is repeat masked due to a combination of a retrotransposon LTR (MLT1E3), partial Alu repeat (AluSx) and a disrupted DNA transposon (Charlie 2a). A short open reading frame (ORF) resides within each monomer spanning 594 bp, and encodes a predicted TATA binding protein associated factor 11 like protein (TAF11-Like). Three SNPs reside within the ORF in the different monomer sequences represented in the hg18 build. None introduce any frame shifts or premature stop codons and therefore maintain the reading frame of the ORF. One SNP is silent exchanging a glycine codon for another (within the ORF a T-C at nucleotide 24 changing a GGT to a GGC). The other two SNPs result in an amino acid change: a T-G at nucleotide 298 changing TCC to GCC and consequently a seriene to alanine (amino acid 99 within the ORF), and a A-G at nucleotide 581 changing a AAA to an AGA resulting in a lysine to arginine change (amino acid 194 in the ORF).

**Figure 2:**
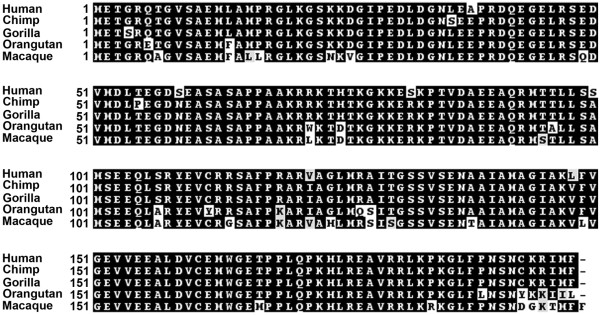
**Conservation of TAF11-Like protein in primates**. Alignment of the predicted amino-acid sequence of the TAF11-Like protein from great apes and macaque. The primate is listed to the left, as is the amino-acid residue number. Identical amino-acids are highlighted in black, amino-acids with similar properties are black letters shaded grey and divergent residues are black letters on a white background. Alignments were prepared using ClustalW2 http://www.ebi.ac.uk/Tools/clustalw2/index.html and the output used to generate the shaded version shown using the Boxshade 3.21 server http://www.ch.embnet.org/software/BOX_form.html. Chimp sequence obtained from *Pan troglodytes*, complete BAC clone containing 20 copies of TAF11-Like (Accession number AC182494). Gorilla sequence obtained from *Gorilla gorilla *genome assembly gorGor3. Orangutan sequence obtained from Pongo pygmaeus genome assembly PPYG2. Macaque sequence obtained from *Macaca mulatta *sequence (Accession number NC_007863).

A comparison of the ORF nucleotide sequence against entries in the nucleotide collection database using BLAST http://blast.ncbi.nlm.nih.gov/Blast.cgi identified a conserved sequence in primates. Translation of these sequences (Figure 2) reveals a conserved ORF of 198 amino acids in great apes (90.9-96.0% identity) and 199 amino acids in Macaque (86.4% identity). Of note, amino acid 99 in the human ORF that is either an alanine or seriene based on the SNPs described above is an alanine in all the primates examined. Likewise, the SNP that results in either an arginine or lysine at amino acid 194 is a lysine in the great apes, but a glycine in Macaque. However, until greater sequence coverage of the TAF11-like array is achieved in all primates, the significance of these coding SNPs cannot be readily assessed at this time. A comparison of the *TAF11-Like *nucleotide sequence against the genomes of each primate revealed for all a tandem array located within a region of synteny to human 5p15 that is in the vicinity of *BASP1*, and therefore likely identifying the orthologous primate macrosatellite arrays.

### Copy number variation, meiotic and mitotic instability of the TAF11-Like macrosatellite array

Assembly of large tandem repeat DNA sequence is problematic for *in silico *assembly, in particular the challenge of whether to align two identical sequences as the same or separate given that *in vivo *they may actually be immediately adjacent to one another. According to the current DNA assembly, the array is largely composed of 17 monomers arranged in tandem, with 3 complete monomers inverted at the distal edge relative to the main array. Fragments of monomers can also be found several kb away from the immediate edges of the array (Figure 1B). To investigate the copy number variation of the TAF11-Like array, genomic DNA from 22 unrelated individuals was embedded in agarose plugs and subjected to digestion with XbaI which does not have any recognition sites within any complete monomers, but is predicted to cut immediately flanking the main array (Figure 1B). The DNA fragments were separated by pulsed field gel electrophoresis (PFGE) and Southern blotted DNA hybridized with a probe specific to the array (Table 1). The size of the hybridizing bands ranged from 34 kb to 335 kb indicating that the size of the TAF11-Like array is very polymorphic in the general population (Figure 1C). Because an individual monomer is approximately 3.4 kb, these data would indicate that as few as 10 and as many as 98 tandem copies of a monomer can be present at an array, a range comparable to that seen for the chromosome 4 macrosatellite array D4Z4.

**Table 1:** Macrosatellite primers

Target	Application	Forward	Reverse	Size
Chr5	Probe	ACTCGTGACGAACCGACTTG	GCTTGTAGGCTTATGTCTGG	571 bp
Chr5	Probe	TGATTCCTCCAGCTGGAAAG	CAGCAAGAAGGATGGAATCC	789 bp
Chr13	Probe	GGAAATCACGGAGACCGCAG	GTTCCATTCCAATCGCCTTG	472 bp
Chr4/19	Probe	GGAGGGTGGGTTTCTTGCAG	AGACGTTGAGCCAGACTAAG	576 bp
Chr9	Probe	GGGCAAATTTCGAGTGAAGG	CCAGTTCCAGCGGTGATTTC	779 bp
Chr5	RT-PCR	CAGGGTTGTCATCCTCTGAG	CAGCAAGAAGGATGGAATCC	226 bp
Chr13	RT-PCR	GGAAATCACGGAGACCGCAG	ACAACGTGGGTGGCAGATAG	306 bp
Chr4/19	RT-PCR	GGGTGGGTTTCTTGCACGTC	CGGGGAAGTCTTCTTTGTGG	594 bp
Chr9	RT-PCR	TGGTTGACGACTCGCGAGAG	CAAAGCACCGGCCTAGATCG	154 bp
GAPDH	RT-PCR	GAAGGTGAAGGTCGGAGTC	AGGTCCACCACTGACACGTT	729 bp

Primers used in the current study to amplify the macrosatellites listed for probe generation or assessment of expression by RT-PCR analysis.

**Figure 3:**
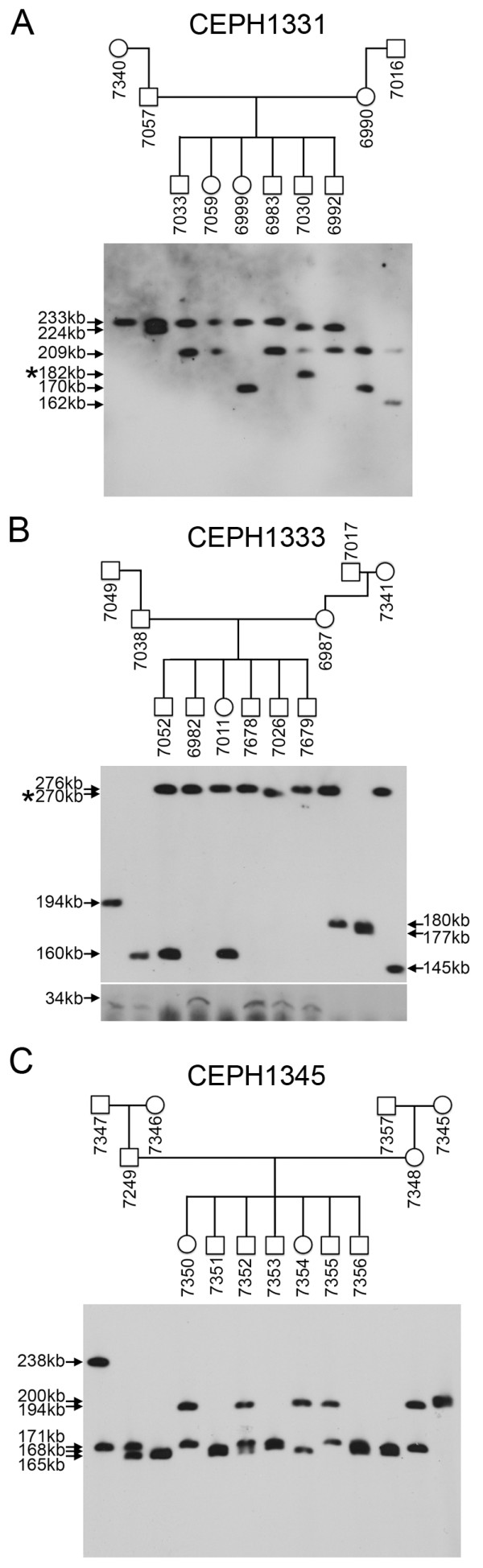
**Unstable transmission of the chromosome 5 macrosatellite**. Inheritance of the chromosome 5 macrosatellite through three generations in three CEPH Utah pedigrees. (A) CEPH-1331, (B) CEPH-1333 and (C) CEPH-1345. Members of each family are indicated above each blot in the pedigrees and the numbers given are the Coriell GM0- ID for each member of the family. The size of the hybridizing fragments are given to the left and right sides of the blots. The blot in image (B) comes from two separate blots for which DNA was separated under different conditions to ensure resolution and visualization of the largest and smallest arrays. The asterisk indicate alleles of altered size.

Given the polymorphic nature of the TAF11-Like array, we investigated the stability of the array through meiosis and mitosis by monitoring Mendelian inheritance of alleles of the array through three generations in three independent CEPH families (Figure 3). Whereas most individuals inherited arrays that were the same size as those of their parents, one individual acquired an allele approximately two repeat units smaller than siblings inheriting the same allele (Figure 3B, 7026) suggesting meiotic instability. A second individual showed the appearance of a new smaller allele as well as retaining the parental allele (Figure 3A, 7030), indicative of somatic instability of the array. To rule out possible contamination of the 7030 cell line with cells from an unrelated individual, alleles of other macrosatellites were assessed on the same blot. In each case, 7030 alleles were consistent with the parents and no additional bands were detected (data not shown).

### Expression of the TAF11-Like array

**Figure 4:**
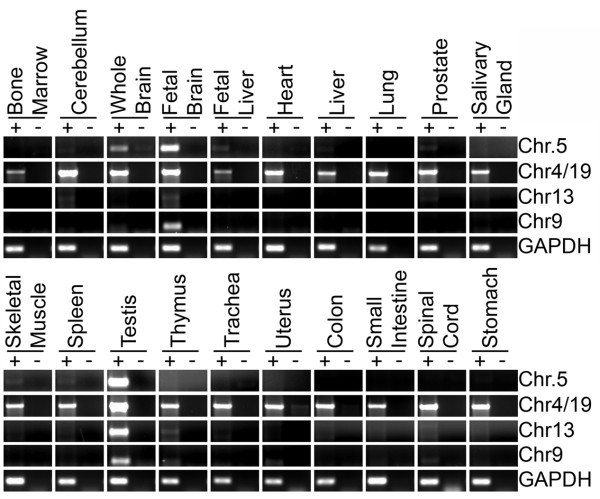
**Expression profile of macrosatellite arrays**. RT-PCR analysis of the macrosatellite arrays listed to the right in a panel of cDNA form 20 different human tissues. The tissues are listed above each agarose gel image with "+" indicating cDNA with reverse transcriptase and "-" indicating the no reverse transcriptase control. Details regarding the size of product and sequences of primers used are given in Table 1.

Given the conserved ORF (Figure 2) and similarity to TAF11, reverse transcription PCR (RT-PCR) was performed on a panel of complementary DNA (cDNA) prepared from total RNA isolated from 20 human tissues. Most tissues did not show evidence of *TAF11-Like *expression with the notable exception of testis, fetal brain and whole brain (Figure 4). Weaker signals could also be detected in fetal liver and prostate.

### Characterization of the chromosome 4 and 19 SST1 macrosatellites

**Figure 5:**
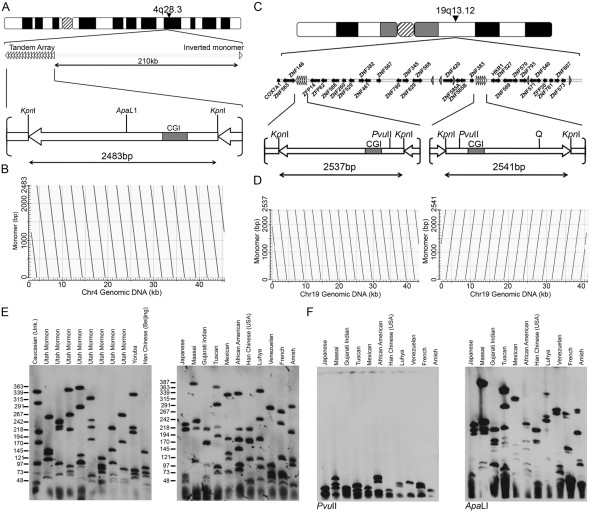
**Characterization of the Chromosome 4 and 19 SST1 macrosatellite arrays**. (A) Ideogram of chromosome 4 and schematic map of the genomic interval around the array, including a single inverted monomer 210 kb distal. Below is a schematic of a 2.4 kb monomer. The CpG island is marked by the grey box (CGI). KpnI and ApaL1 restriction endonuclease sites are indicated. (B) The y-axis is a 2.4 kb monomer and the x-axis is the 48 kb genomic interval (132.8 Mb hg18). Dot-plot generated using NCBI Blast, and output image labelled in Adobe Photoshop CS2. (C) Ideogram of chromosome 19 and schematic map of 1.6 Mb genomic interval encompassing the arrays. Partial monomers represented by single arrow heads. Black arrows represent genes and direction/extent of transcription. Below is a schematic of an individual 2.5 kb monomer. KpnI and PvuII restriction endonuclease sites are indicated. (D) The y-axis is a single 2.5 kb monomer and the x-axis is the 45 kb (41.4 Mb hg18) or 41 kb (42.4 Mb hg18) genomic intervals. (E) Copy number variation of the chromosome 4/19 macrosatellite arrays. Southern of XbaI cut DNA from 22 unrelated individuals separated by PFGE hybridized with a chromosome 4/19 macrosatellite probe. The ethnicity of the individuals used is indicated at the top. Size markers are given along the left edges in kb. (F) Southern of 11 unrelated individuals genomic DNA cut with either PvuII or ApaLI hybridized with a chromosome 4/19 macrosatellite probe.

Along with the TAF11-Like array, a macrosatellite was identified on chromosome 4 and 19 (Figure 5). This array was previously reported as a moderately repetitive tandem array that was initially identified as a repetitive sequence with homology to adenovirus [33]. The repeat was named SST1 due to the restriction enzyme SstI cutting once per monomer generating a common 2.5 kb fragment on Southern hybridization. On chromosome 4 the SST1 array resides within a gene poor region of 4q28.3 with the nearest annotated flanking genes being *PCDH10 *1.4 Mb distal to the array and *C4orf33 *approximately 2.6Mb proximal. The array is composed of 2.4kb monomers in tandem with one partial monomer inverted and 210 kb distal to the main array (Figure 5A). Each monomer is moderately GC rich (64%) and contains a CpG island (CGI) of about 400 bp. With the exception of a few simple repeats accounting for less than 5% of a monomer, the remaining sequence does not contain any other repetitive elements. A comparison of the 2.4-kb sequence to entries in the public databases primarily indentifies other chromosome 4 monomers (97-100% sequence identity) as well as monomers from the chromosome 19 arrays (92-93% sequence identity). Other matches of >90% over 1.8 kb were identified on chromosome 9 (4 matches) and 21 (single match). In the current DNA assembly, the chromosome 4 SST1 array is represented by 17 uninterrupted 2.4 kb monomers therefore covering approximately 40 kb (Figure 5B).

Chromosome 19 contains two SST1 arrays separated by approximately 1 Mb with several partial monomers scattered either side of the more distal array (Figure 5C). Unlike the chromosome 4 array, on chromosome 19 the two SST1 macrosatellites are embedded within a gene rich region of 19q13.12. Intriguingly, most of the genes encode members of the kruppel C2H2-type zinc-finger protein family with two genes proximal, sixteen between and eleven distal of the two SST1 macrosatellites. Monomers of the chromosome 19 SST1 arrays are slightly longer than those at chromosome 4 (2.5 kb v 2.4 kb). Within the proximal or distal chromosome 19 array monomers share >99% sequence identity compared to 97-98% identity between the two arrays based on BLAT results using a single monomer from each array. The orientations of the two arrays are inverted relative to one another and are represented by 17 (proximal array) and 15 monomers (distal array) arranged in tandem (Figure 5D).

### Copy number variation and instability of the SST1 macrosatellite arrays

To investigate copy number variation of monomers within the SST1 macrosatellite arrays, agarose embedded DNA from 22 unrelated individuals was digested with XbaI that does not cut within the array and separated by PFGE before Southern blotting and hybridization with a probe specific to the SST1 arrays. Given the high sequence identity between the three arrays, the probe detected both chromosome 4 and 19 SST1 macrosatellites with most individuals showing all 6 alleles (Figure 5E). Alleles ranged from as large as 387 kb to less than 35 kb indicating tandem arrays of between 14 to 154 monomers. In order to determine if large and small alleles are common to both chromosome 19 and/or chromosome 4 SST1 macrosatellite arrays, PFGE was performed on agarose embedded DNA digested with enzymes that specifically cut each monomer within the chromosome 4 array (ApaLI) or chromosome 19 arrays (PvuII) (Figure 5A and 5B). Specifically removing the chromosome 19 arrays with PvuII resulted in small alleles only, whereas most large alleles remained intact and comparable in size upon digestion with ApaLI that specifically removes the chromosome 4 arrays (Figure 5F). These data suggest that large SST1 arrays are more common on chromosome 19 than chromosome 4.

**Figure 6:**
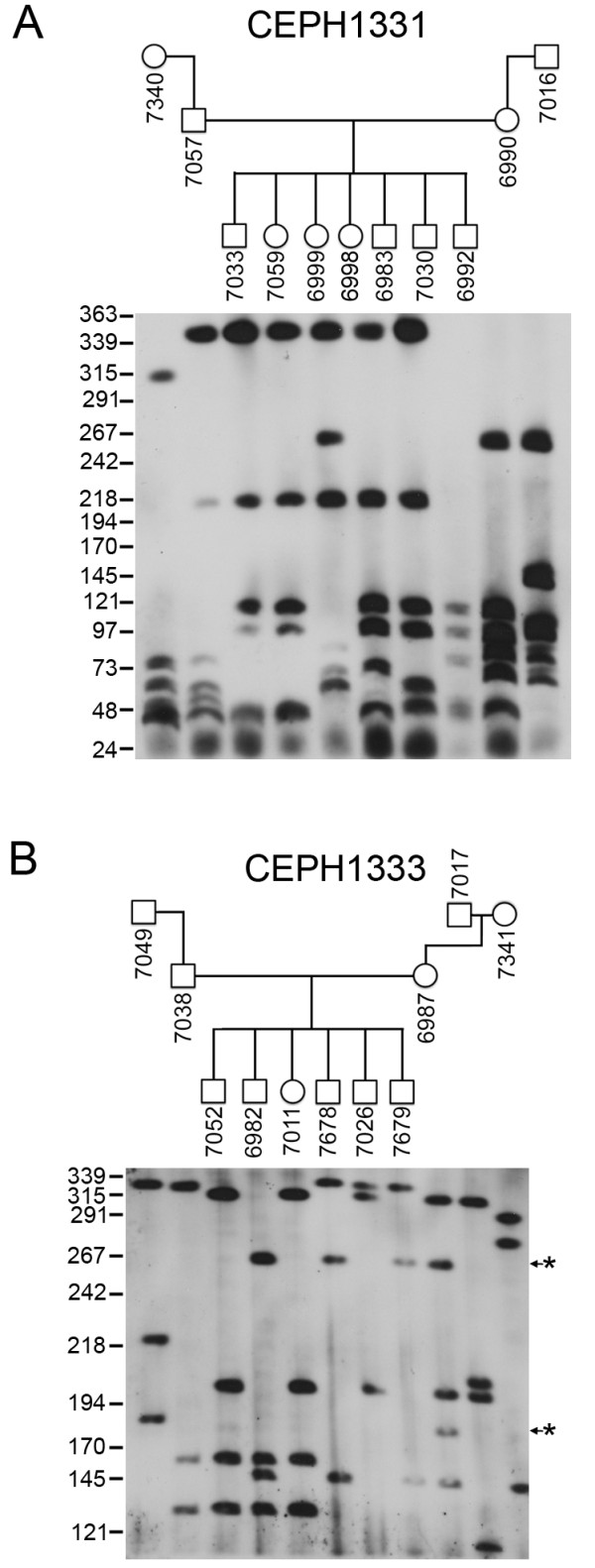
**Transmission of the chromosome 4 and 19 macrosatellite arrays**. Inheritance of the chromosome 4 and chromosome 19 macrosatellite arrays through three generations in two CEPH Utah pedigrees. (A) CEPH-1331 and (B) CEPH-1333. Members of each family are indicated above each blot in the pedigrees and the numbers given are the Coriell GM0- ID for each member of the family. Size markers are given along the left edges in kb. The asterisk indicate alleles of altered size.

Given the large range of allele sizes for the SST1 arrays, we next investigated stability of transmission of alleles through three generations in two CEPH families (Figure 6). In one individual (Figure 6B, 6987), two new alleles of approximately 260 kb and 180 kb can be detected that are not present in either parent. The larger of the two alleles was stably transmitted through the germ line to three offspring, whereas the smaller allele was not detected.

### Expression of the SST1 array

According to the UCSC genome browser, a cluster of ESTs match a monomer centered upon the CGI. Primers were designed to amplify across the CGI and were used to detect expression from the SST1 macrosatellite using cDNA prepared from 20 human tissues. Expression was readily detected in all tissues (Figure 4, second row). Several open reading frames exist within chromosome 4 and 19 monomers, but none match any known proteins in the public databases.

### Characterization of the PRR20 macrosatellite on chromosome 13

**Figure 7:**
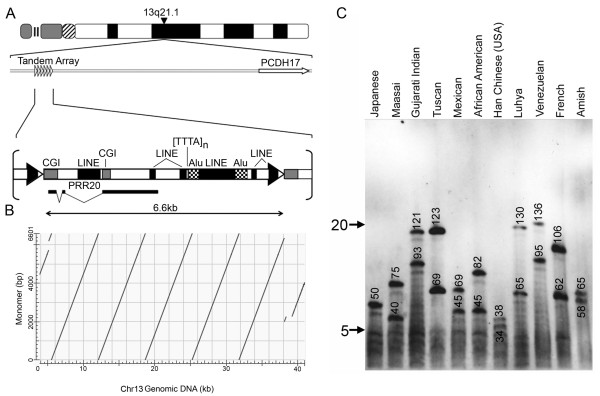
**Characterization of the chromosome 13 macrosatellite array**. (A) Ideogram of chromosome 13 and schematic map of the genomic interval indicating *PCDH17 *450 kb distal. The array is represented by the right facing open arrow heads. Below is a schematic representation of a single 6.6 kb monomer. The location and gene structure of the transcript *PRR20 *is indicated below the monomer transcribing from left to right (proximal to distal). LINE, Alu and TTTA-repeat are indicated by the labelled shaded boxes, as are the two CGIs. (B) Higher-order organization of the chromosome 13 array. A single 6.6 kb monomer sequence is on the y-axis, whereas the ~41 kb genomic interval is on the x-axis (57.1 Mb hg18). Dot-plot generated using NCBI Blast, and output image labelled in Adobe Photoshop CS2. (C) Copy number variation of the chromosome 13 macrosatellite array. Southern blot analysis of EcoRI cut DNA from 11 unrelated individuals separated by PFGE and hybridized with a chromosome 13 macrosatellite probe. The ethnicity of the individuals used is indicated at the top. The size of the hybridizing fragments in kb are indicated on the blot. The arrows to the left indicate the range in size of the array as represented by monomer copy number, with 5 in the smallest array and approximately 20 in the largest array.

**Figure 8:**
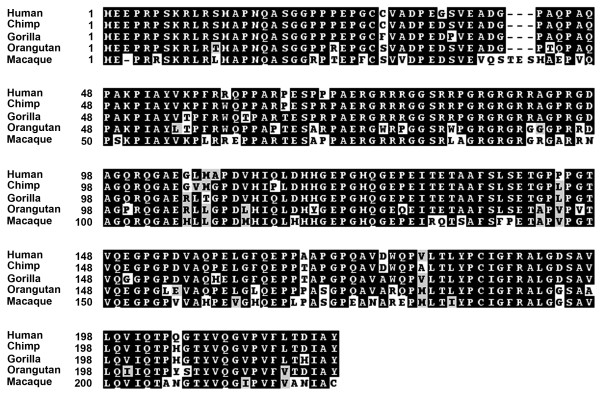
**Conservation of PRR20 in primates**. Alignment of the predicted amino-acid sequence of the PRR20 protein from great apes and macaque. The primate is listed to the left, as is the amino-acid residue number. Identical amino-acids are highlighted in black, amino-acids with similar properties are black letters shaded grey and divergent residues are black letters on a white background. Gaps in the alignment are represented by dashes. Alignments were prepared using ClustalW2 http://www.ebi.ac.uk/Tools/clustalw2/index.html and the output used to generate the shaded version shown using the Boxshade 3.21 server http://www.ch.embnet.org/software/BOX_form.html. Chimp sequence obtained from Pan troglodytes genome assembly (CGSC 2.1/panTro2) using the UCSC genome browser http://genome.ucsc.edu/. Gorilla sequence obtained from Gorilla gorilla genome assembly (gorGor3) using the Ensembl genome browser http://www.ensembl.org/index.html. Orangutan sequence obtained from a complete *Pongo abelii *BAC clone (Accession Number AC206922). Macaque sequence obtained from *Macaca mulatta *genome assembly (rheMac2) using the UCSC genome browser.

A third candidate macrosatellite array was detected at chromosome 13q21.1. The array resides within a gene poor region with the nearest annotated gene 450 kb distal (*PCDH17*) and over 4 Mb proximal (*OLFM4*)(Figure 7A). An individual tandem repeat unit is 6.6 kb, is approximately 50% GC and 43% repeat masked for LINE, Alu and a simple repeat. Two CGIs reside within a single monomer covering 333 bp and 201 bp. Each monomer contains the proline-rich-20 gene (*PRR20*). The gene is composed of three exons of 214 bp, 71 bp and 1562 bp and encodes a 221 amino acid proline rich protein. RT-PCR analysis indicates that PRR20 is primarily expressed in the testis with low levels of transcript detected in cerebellum, fetal brain, prostate and thymus (Figure 4, 3^rd ^row). Orthologous copies of *PRR20 *were identified in great apes and *Macaca mulatta *that encode PRR20 proteins that have 95-71% amino acid identity (chimp to macaque) with human PRR20 (Figure 8). Furthermore, in the great apes, *PRR20 *is arranged into a tandem array as seen in humans, with at least one complete orangutan (Pongo abelii) BAC clone (Accession Number AC206922) containing 17 copies of the gene arranged in tandem (Data not shown). Of the five complete human PRR20 monomers present in the human genome, no SNPs exist within the ORF between the different monomers.

According to the human genome sequence, the five complete monomers are arranged in tandem (Figure 7B). We sought to confirm the size of the array and investigate copy number variation in 11 unrelated individuals. Alleles for the PRR20 array ranged from 5 tandem copies to as many as 20 (Figure 7C) indicating that this is indeed a polymorphic macrosatellite.

### Characterization of the ZAV macrosatellite array on human chromosome 9q32

**Figure 9:**
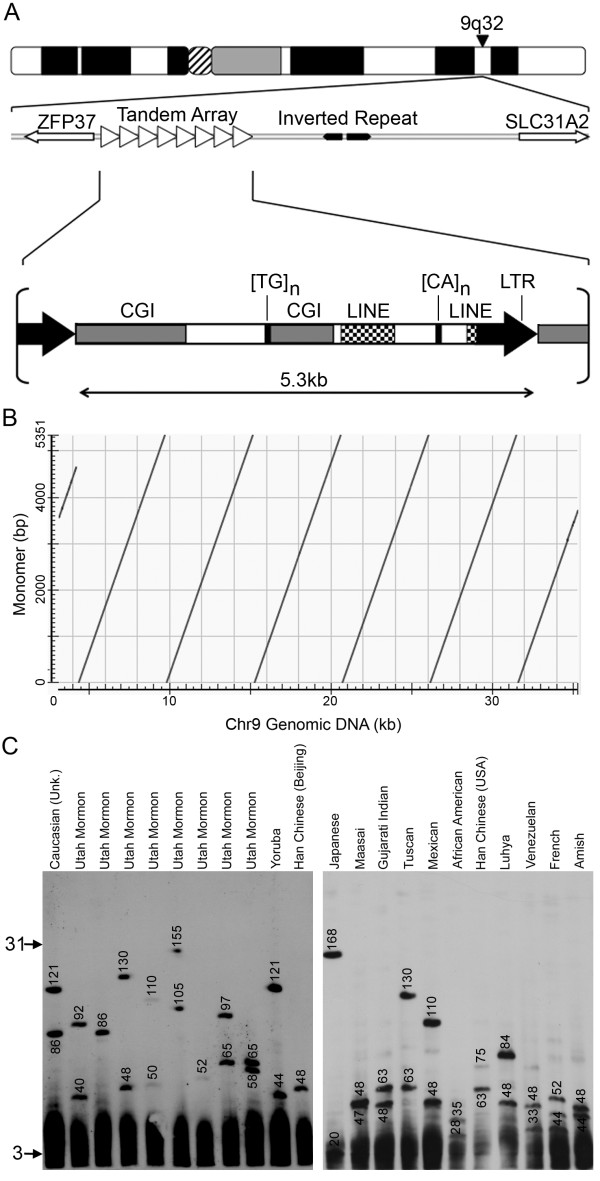
**Characterization of the chromosome 9 ZAV macrosatellite array**. (A) Ideogram of chromosome 9 and schematic map of the genomic interval. The array is represented by the right facing open arrow heads. Immediately proximal (<2 kb) is the *ZFP37 *gene. To the distal side of the array is a inverted repeat and the *SLC31A2 *gene (60 kb distal). Below is a schematic representation of a single 5.3 kb monomer. LINE & LTR regions are highlighted as is the microsatellite repeats and the two CG1s. (B) Higher-order organization of the chromosome 9. A single 5.3 kb monomer sequence is on the y-axis, whereas the ~35 kb (114.8 Mb hg18).. Dot-plot generated using NCBI Blast, and output image labelled in Adobe Photoshop CS2. (C) Copy number variation of the chromosome 9 macrosatellite array. Southern of XbaI cut DNA from 22 unrelated individuals separated by PFGE and hybridized with a chromosome 9 macrosatellite probe. The ethnicity of the individuals used is indicated at the top. The size of the hybridizing fragments in kb are indicated on the blot. The arrows to the left indicate the range in size of the array as represented by monomer copy number, with 3 in the smallest array and approximately 31 in the largest array.

One other macrosatellite candidate identified in our search resides within a moderately gene rich region of human chromosome 9q32 (Figure 9A). The closest gene is *ZFP37*, a member of the KRAB zinc finger gene family that is located immediately adjacent to the array within 2 kb of the proximal edge and is transcribed away from the array. On the distal side the nearest annotated gene is *SLC31A2 *which is 62 kb away from the edge of the array. Between the array and *SLC31A2 *is an inverted repeat that transcribes a non-coding RNA of unknown function. A similar inverted repeat is present upstream of the mouse *Zfp37 *gene on chromosome 4qB3, but there is no evidence of the tandem array suggesting that this genomic feature is a more recent acquisition in humans (data not shown).

Each tandem repeat unit of the array is 5.3 kb and 60% GC. A little over 28% of each monomer is repeat masked due to the presence of simple microsatellite repeats, an LTR and regions of LINE homology. Two CGIs are found in each repeat unit, one spanning 1.4 kb and the other close to 700 bp. Therefore almost 40% of each monomer, accounting for most of the non-repeat masked region, is a characterized by a CGI. In the current build of the human genome, the array is annotated as 5 repeat units arranged in tandem (Figure 9B). As for the other arrays we examined 22 unrelated individuals to see if the array was polymorphic. Alleles for the array ranged from as small as 20 kb to 168 kb (Figure 9C) indicating that this is indeed a macrosatellite repeat with between 3-31 tandem repeat units and was termed the *ZFP37*-Associated VNTR (ZAV).

One EST aligned with the larger of the two CGIs. We designed primers to this region and looked to see if the array is expressed in any of the 20 human tissue cDNA samples. Clearly the ZAV array is expressed in fetal brain and testis, with weaker signals in whole brain, thymus, uterus and spinal cord (Figure 4, fourth row).

## Discussion

Here we describe the organization, size variation and expression of four macrosatellite arrays in the human genome that is summarized in Table 2. Consistent with other macrosatellite arrays [3-6] alleles of each array show a wide range of tandem repeat unit copy number variation, with the chromosome 19 SST1 macrosatellite displaying alleles with the largest number of tandem repeat units described to date for a human macrosatellite. Also consistent with data for the RS447 macrosatellite [3] we present evidence of meiotic and mitotic instability for the SST1 and TAF11-Like arrays in a relatively small sample size. Of note, the chromosome 9q32 ZAV macrosatellite described here and the RS447 array reside within common fragile sites in the human genome [34] and could potentially contribute to chromosome breaks and rearrangements frequently observed in tumor cells. Taken together, these data are consistent with the reported instability of macrosatellite sequences and further highlight their contribution as extreme forms of copy number variation in the human genome.

**Table 2:** Macrosatellite Summary

Array	TAF11-Like	SST1 (Chr4 & 19)	PRR20	ZAV
Monomer size	3.4 kb	2.4-2.5 kb	6.6 kb	5.3 kb
GC Content	50%	64%	50%	60%
Number of unrelated array alleles	54	134	20	41
Average array allele size (Median)	178 kb (178 kb)	161 kb (127 kb)	76 kb (67 kb)	71 kb (58 kb)
Range allele size	34-335 kb	35-387 kb	34-136 kb	20-168 kb
Inferred monomer copy number	10-98	14-154	5-20	3-31
Expression (weaker)	Testis Fetal Brain Whole Brain (Fetal Liver & Prostate)	All tissues examined	Testis (Cerebellum, Fetal Brain, Prostate & Thymus)	Testis Fetal Brain (Whole Brain, Thymus, Uterus & Spinal Cord)
ORF	TAF11-Like	-	PRR20	-

Table summarizing features of the macrosatellite arrays

Among the macrosatellites described here, the SST1 array is the only one that is not chromosome specific, but has arrays of significant size 1 Mb apart on 19q13.2 and an additional highly conserved array on chromosome 4q28.3 [2,33]. A similar situation is true for the FSHD-associated array D4Z4 that has near identical arrays on chromosomes 4q35 and 10q26. Exchange between the 4q and 10q D4Z4 arrays is common in the general population [35]. The subtelomeric location of the arrays results only in the expansion or contraction of an array as well as exchange of the limited DNA sequence from the distal edge of the array to the telomere. Should the chromosome 4 and 19 SST1 arrays recombine this would result in an extensive exchange. While we are aware of no such chromosome 4:19 translocations, it remains a distinct possibility that the two inverted chromosome 19q13 SST1 arrays could undergo intra-chromosomal non-allelic homologous recombination inverting the 1 Mb interval between the arrays, especially given the extensive size and >97% sequence identity between the two macrosatellite arrays.

An independent investigation using array comparative genome hybridization identified contracted alleles of the TAF11-Like array as a possible contributor to schizophrenia [36]. In one group of families, small alleles (<21 tandem repeat units) segregated with schizophrenia with only one unaffected sibling carrying a small sized allele. However, this association did not extend to other schizophrenia families and therefore linkage of small TAF11-Like arrays to the disease is not statistically significant. Interestingly, the extended analysis to other schizophrenia families made use of quantitative PCR (qPCR) as opposed to PFGE to assess allele size. As the authors acknowledge, the results therefore represent the combined tandem repeat content of both chromosome 5 arrays, potentially masking small alleles. In the present study we also identified small alleles of the TAF11-Like array, with the smallest (~10 tandem repeat units) comparable in size to the pathogenic contracted D4Z4 alleles in FSHD [5]. For these particular individuals, who have alleles of 10 and 81 tandem repeats, qPCR would have indicated approximately 50 tandem repeat units in each chromosome 5 array. This highlights that despite numerous advances in genome research tools, PFGE remains the most effective and reliable means to measure allele size of tandem repeat DNA.

Expression analysis of the four macrosatellite described here revealed that all of the arrays are expressed at high levels in the testis and to a lesser extent in fetal brain and in adult brain (except PRR20). The notable exception is SST1 that is ubiquitously expressed in all tissues examined. Furthermore, expression was independent of a clear ORF as demonstrated by the SST1 and ZAV macrosatellites, although it remains a possibility that either has a short ORF with no homology to known proteins. Expression in the testis is a defining feature of the cancer/testis (CT) genes [37], many of which are arranged into tandem arrays [38,39]. Therefore, it is possible that the ZAV, PRR20 or TAF11-Like macrosatellites are novel members of the cancer/testis group, as some CT-genes are expressed in developing neurons as well as testis [40]. Regardless, the strong expression in testis indicates that these arrays are likely packaged into an alternate chromatin state in this organ, most likely in the germ cells, as is the case for some MAGE and GAGE CT genes [41].

What influence variation in the number of copies of individual tandem repeat units has, if any, on flanking gene expression remains an important question to be addressed, despite the conflicting data regarding D4Z4 [11-19]. For some such as the chromosome 4 SST1 array or the PRR20 array on chromosome 13q21.1 any influence is likely negligible given that the nearest annotated genes are considerable physical distances away. The TAF11-Like array is also some distance from the distal BASP1 gene (210 kb) and therefore is also unlikely to exert any influence on its expression. However, this is making the assumption that influence is 2-dimensional and does not take into account where in 3-dimensional space the arrays are located relative to other genes. In contrast, the 9q32 ZAV array and SST1 arrays at 19q13.12 could conceivably directly influence flanking gene expression. The ZAV array is approximately 2 kb from the proximal *ZFP37 *gene is composed of GC rich tandem repeat units that each contain two extensive CGIs. Evidence supporting such a notion comes from the fact that the murine homolog *Zfp-37 *is expressed specifically in the brain and testis [42], mirroring the human array expression pattern. Therefore, it is tempting to posit that the array in humans is an extensive regulatory element of the ZFP37 gene. The location of the chromosome 19 SST1 arrays are intriguing due to the presence of the 29 zinc-finger genes that reside between and immediately proximal/distal of the two macrosatellites. Of the approximately 800 zinc-finger genes in the human genome, a disproportionate number reside on chromosome 19, with most residing in one of 11 clusters [43]. This is not the only kruppel-type zinc finger cluster on chromosome 19 that is associated with tandem repeat DNA, as an extensive cluster at 19p12 is interspersed with blocks of β-satellite [44]. Kruppel-type zinc fingers are implicated in transcriptional regulation of many crucial processes from development to control of the cell cycle to pluripotency [45]. Despite their close proximity, zinc finger genes within a cluster are differentially expressed [46] and therefore intricate modes of regulation must be required to tightly regulate the expression of cluster members. We propose that the SST1 array at 19q13.12 has a role in the regulation of the surrounding zinc finger gene cluster.

## Conclusions

The wealth of information made available from the human genome project has provided an invaluable tool to assist in the identification and characterization of complex repetitive DNA elements such as the macrosatellites. Our results more than double the number of characterized macrosatellite arrays in the human genome and further highlight our lack of understanding as to the role of these unusual sequences in genome biology despite their direct relevance to disease. We anticipate that further characterization of these arrays will reveal the functional significance of macrosatellites arrays, explaining why they are retained in our genome.

## Methods

### Cell lines

Lymphoblastoid cell lines of CEPH family members and the individuals used in the variation panels were obtained from the Coriell Institute for Medical Research http://www.coriell.org/. Cells were maintained according to Coriell recommendations. Culture media (RPMI), fetal bovine serum and supplements were all obtained from Invitrogen corp.

### Plug preparation

Approximately 4 × 10^7 ^cells were resuspended in 1 ml of L-buffer (100 mM EDTA [8.0], 10 mM Tris-HCl [8.0], 20 mM NaCl), before mixing 1:1 with 1.0% (w/v) molten low-melt agarose (Biorad). The cell mixture was transferred to plug moulds (Biorad) with ~80 ul of the cell suspension per plug (approximately 1.6 × 10^6 ^cells/plug). Plugs were allowed to set at 4°C for 10 minutes before transfer to 10 volumes of L-buffer containing 1% (w/v) sarkosyl and 1 mg/ml Proteinase-K (Roche) and incubating overnight at 50°C. Plugs were rinsed with water before three washes of one hour each with 50 volumes of TE [8.0]. Plugs were incubated at 50°C for 30 minutes in 10 volumes of TE [8.0] supplemented with 80 ug/ml PMSF (Roche). Plugs were rinsed once more with water before three additional hour-long washes in 50 volumes of TE [8.0] at room temperature before storage at 4°C.

### Pulsed field gel electrophoresis

Agarose embedded DNA was digested with the restriction enzymes given in the legend for the appropriate data figures. All enzymes were obtained from NEB. Each plug was first equilibrated in 300 ul of 1× digest buffer at room temperature for 20 minutes, before replacement of buffer with 100 ul of 1× digest buffer containing 200 units of restriction enzyme. Digests were performed overnight at 37°C. Plugs were loaded onto a 1.0% agarose gel prepared using pulsed field certified agarose (Biorad) in 0.5 × TBE. DNA was separated at 14°C in 0.5 × TBE according to the time and conditions determined by the auto algorithm function of the CHEF Mapper (Biorad). Markers were loaded in the outer lanes (NEB, MidRange PFG Markers I and II).

### Southern blotting & hybridization

At the end of the PFGE run, the gel was rinsed with water before staining with ethidium bromide (1 ug/ml) at room temperature for 30 minutes. The gel was washed twice with water for 15 minutes each and an image captured. The gel was then treated with 0.25 M HCl for 15 minutes before denaturing for 30 minutes (1.5 M NaCl, 0.5 M NaOH). DNA was transferred to Hybond-N+ (GE Healthcare) overnight by standard Southern blotting [47]. The membrane was rinsed with 2 × SSC before baking at 120°C for 30 minutes.

Macrosatellite specific probes were prepared by PCR amplification using primers listed in table 1. The PCR products were cleaned (Qiagen) before labelling with DIG-11-dUTP by random priming (Roche). The probes were tested for specificity and detection of the anticipated DNA fragment size on a Southern blot of EcoRI digested total genomic DNA.

Hybridization was performed overnight at 60°C using Expresshyb (Clontech). Blots were washed the following day at 60°C using two 8 minute washes in 2 × SSC, 0.1%SDS followed by one wash of 8 minutes in 0.2 × SSC, 0.1%SDS. The probe was detected using anti-DIG-alkaline phosphatase, blocking, wash and detection buffers according to the manufacturers instructions (Roche). Signals were detected by exposure to photographic film (Kodak).

### Reverse Transcription PCR

Human tissue total RNA was obtained from Clontech (636643). Residual genomic DNA was removed by pre-treating the RNA with DNaseI (Invitrogen) for 20 minutes at room temperature, before heat inactivating the DNaseI at 70°C in the presence of 2.5 mM EDTA for 15 minutes. Complementary DNA was prepared using 1 ug of total RNA with or without MMLV reverse transcriptase (Invitrogen) according to the manufacturers instructions.

cDNA was amplified using Taq polymerase (NEB) with the following cycle: 95°C for 2 minutes, followed by 35 cycles of 95°C 20 seconds, 58°C 20 seconds, 72°C 30 seconds. Primers used for amplification and size of anticipated product are listed in Table 1.

## Competing interests

The authors declare they have no competing interests.

## Authors' contributions

BPC conceived of the study, analyzed and interpreted data, performed experiments, and wrote the manuscript. DCT, GA, SM and BPC carried out experiments. All authors have read and approved the final manuscript.
